# Statistical Enrichment Analysis of Samples: A General-Purpose Tool to Annotate Metadata Neighborhoods of Biological Samples

**DOI:** 10.3389/fdata.2021.725276

**Published:** 2021-09-16

**Authors:** Thanh M. Nguyen, Samuel Bharti, Zongliang Yue, Christopher D. Willey, Jake Y. Chen

**Affiliations:** ^1^ Informatics Institute, School of Medicine, The University of Alabama at Birmingham, Birmingham, AL, United States; ^2^ Centre for Computational Biology and Bioinformatics, Amity Institute of Biotechnology, Amity University, Noida, India; ^3^ Department of Radiation Oncology, School of Medicine, The University of Alabama at Birmingham, Birmingham, AL, United States

**Keywords:** sample enrichment analysis, clinotype, SEAS, glioblastoma multiforme, patient-derived xenograft, patient-derived xenograft

## Abstract

Unsupervised learning techniques, such as clustering and embedding, have been increasingly popular to cluster biomedical samples from high-dimensional biomedical data. Extracting clinical data or sample meta-data shared in common among biomedical samples of a given biological condition remains a major challenge. Here, we describe a powerful analytical method called Statistical Enrichment Analysis of Samples (SEAS) for interpreting clustered or embedded sample data from omics studies. The method derives its power by focusing on sample sets, i.e., groups of biological samples that were constructed for various purposes, e.g., manual curation of samples sharing specific characteristics or automated clusters generated by embedding sample omic profiles from multi-dimensional omics space. The samples in the sample set share common clinical measurements, which we refer to as “clinotypes,” such as age group, gender, treatment status, or survival days. We demonstrate how SEAS yields insights into biological data sets using glioblastoma (GBM) samples. Notably, when analyzing the combined The Cancer Genome Atlas (TCGA)—patient-derived xenograft (PDX) data, SEAS allows approximating the different clinical outcomes of radiotherapy-treated PDX samples, which has not been solved by other tools. The result shows that SEAS may support the clinical decision. The SEAS tool is publicly available as a freely available software package at https://aimed-lab.shinyapps.io/SEAS/.

## Introduction

Systematic software platforms to organize large metadata and clinical data [also called “clinotype” (Nguyen et al., 2021)] is essential in biomedical research (Burgun and Bodenreider, 2008; Ohmann and Kuchinke, 2009). These software platforms, such as (Ta et al., 2018; Kim et al., 2019; Hume et al., 2020), have two key objectives. First, it allows the biomedical researcher to perform manual cohort selection quickly. Here, the researcher inputs the filtering query and gets the data from all patients meeting the filtering criteria. Second, it allows quick data exploration, including data visualization and simple aggregated analysis. Here, the researcher may view the basic characteristic of the selected subcohort, find potential clinical bias, and adjust the filtering criteria to obtain a better subcohort. Integrating Biology and the Bedside (Murphy et al., 2010) is a typical example of a clinical metadata software system. Some systems and techniques may offer more in-depth and specific analysis. For example, Weng et al. (2017) implemented a machine-learning based system to estimate the patients’ cardiovascular risk from the routine checkup records. Fang et al. (Fang et al., 2014) implemented a visual analytic system to view patient’s geographical demographic and disease comorbidities.

On the other hand, the state-of-the-art clinical data software still has three limitations. First, the simple aggregated analysis has not been well-developed for categorical clinical attributes. Therefore, the researcher may not easily find whether a specific categorical attribute is explicit for the selected cohort compared to the whole population. Second, methods to quantify and visualize patients’ similarities have not been implemented. Therefore, the existing clinical software is likely ineffective in clinical support scenarios such as “finding the clinical outcome data about previous patients that are the most similar to the under-treatment patients”. Third, the existing software does not support patient clustering. Therefore, they may not automatically recommend subcohort to the researcher. This feature could provide new insights to biomedical research; for example, a tool that quickly shows two clusters in a treatment-selected cohort may enable a new hypothesis about the treatment outcome.

This work introduces Statistical Enrichment Analysis of Samples (https://aimed-lab.shinyapps.io/SEAS/), a software tool with both online and standalone versions to tackle the above limitations. SEAS graphical user interface is user-friendly, where the user interacts by uploading datafile, primarily uses mouse operations, and requires a very limited amount of typing. Furthermore, SEAS implements methods to analyze numerical and categorical data, compute patient similarity, and automatically cluster the patients. For the demo, we use SEAS to analyzing the glioblastoma multiforme (GBM) patients’ clinical metadata in The Cancer Genome Atlas Program (TCGA) (Verhaak et al., 2010) and estimate the clinical outcome of patient-derived xenograft (PDX) models data.

## SEAS Functions

**FIGURE 1:**
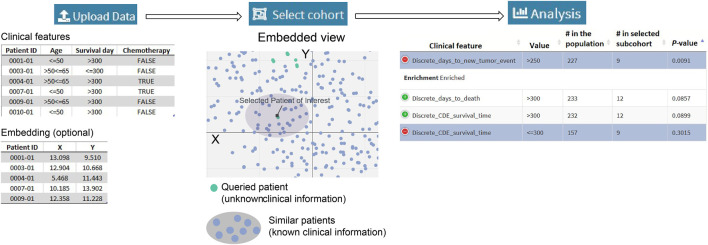
Overview of data processing and analysis.

Figure 1A summarizes a SEAS session. The required input is the clinical metadata that is organized in one table. The user may choose to let SEAS automatically compute and represent the patients’ similarity in a 2D embedding space or optionally upload another patients’ scatterplot. Here, each plot represents a patient, and the distance among the plots should represent patients’ similarities. Then, the user may manually enter a subcohort, automatically let SEAS select a subcohort, or semi-automatically choose a subcohort. After selecting a subcohort, SEAS performs clinical feature enrichment analysis (CFEA) and reports all enriched features in the selected subcohort.

### Automatically Compute Patients’ Similarity and Embedding

In this step, the categorical clinical attributes are digitized as in (Zaki et al., 2014). For example, if the categorical attribute X has three discrete values: low, normal, and high, it can be decomposed into three binary attributes: is_X_low, is_X_normal, is_X_high. If a patient has a “high” categorical value for X, then the patient’s digital representation is (0, 0, 1). On the other hand, the numerical attributes are normalized using the z-score approach.

**FIGURE 2:**
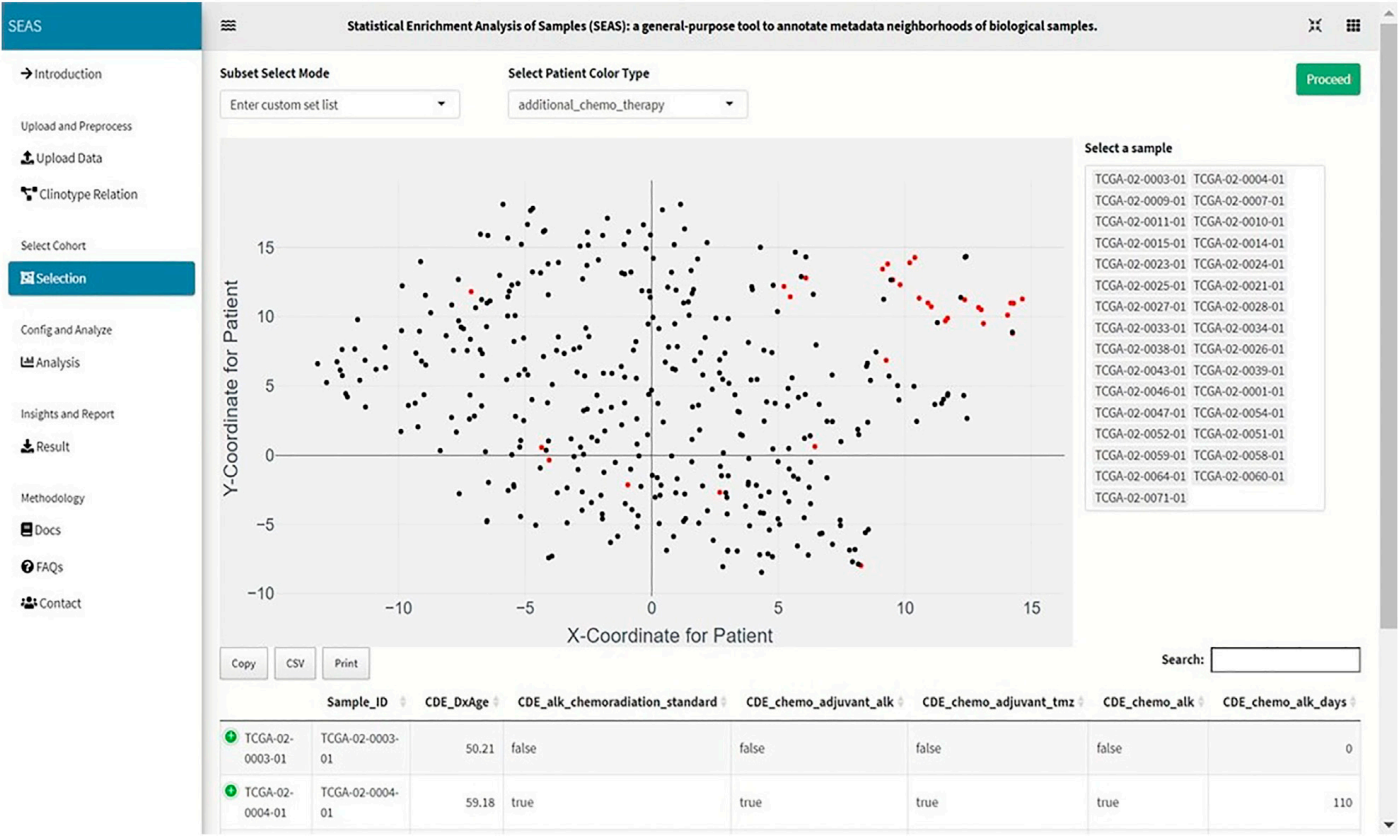
Screenshot showing that SEAS visualizes the TCGA-GBM patients using embedding, and the user manually selects the subcohort.

**FIGURE 3:**
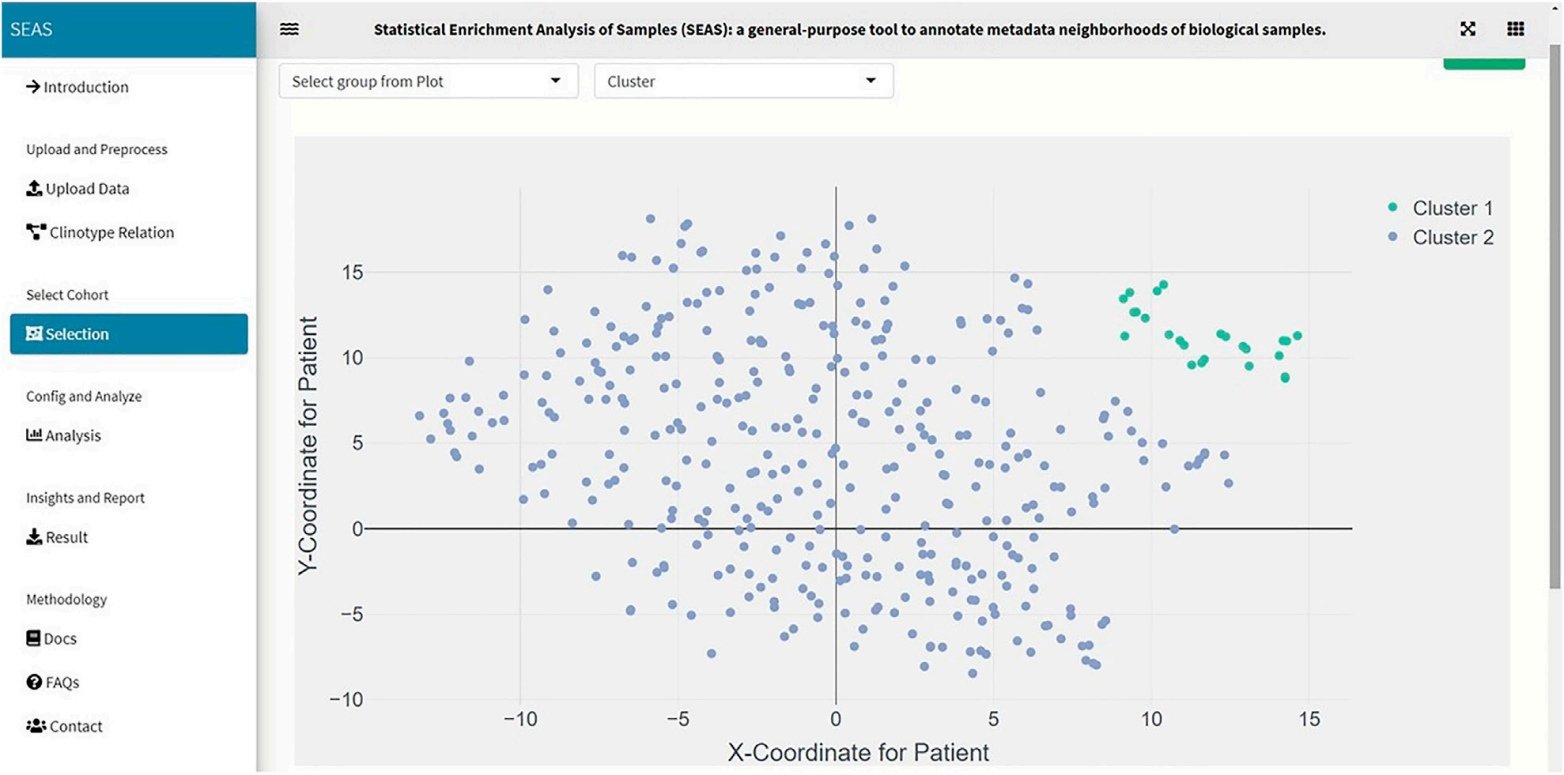
SEAS identifies a subcohort by clustering the TCGA-GBM patients (green dots on the top-right of the embedding scatterplot).

**FIGURE 4:**
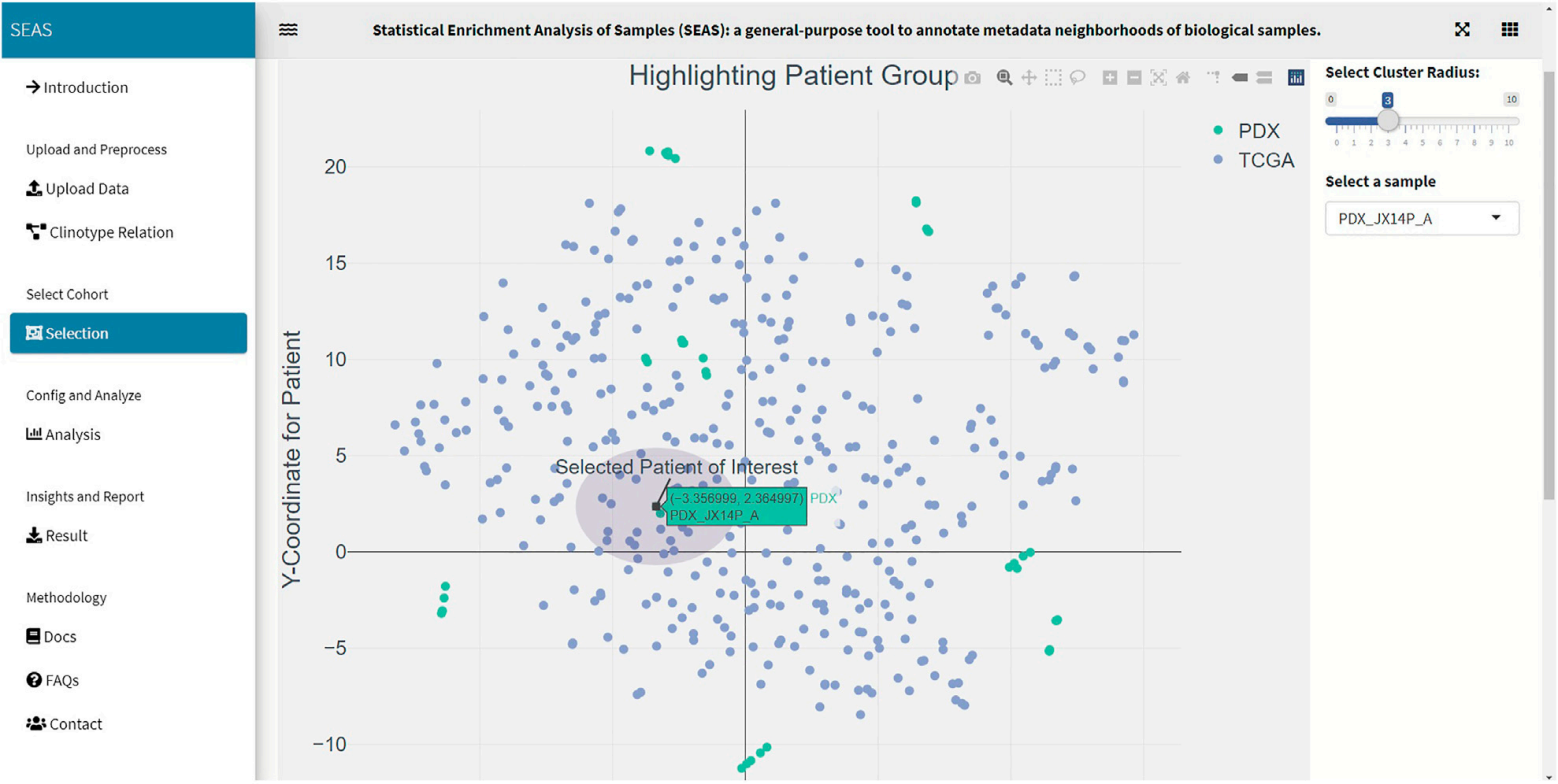
SEAS identifies a subcohort by a circle region around PDX JX14P_A datapoint.

**FIGURE 5:**
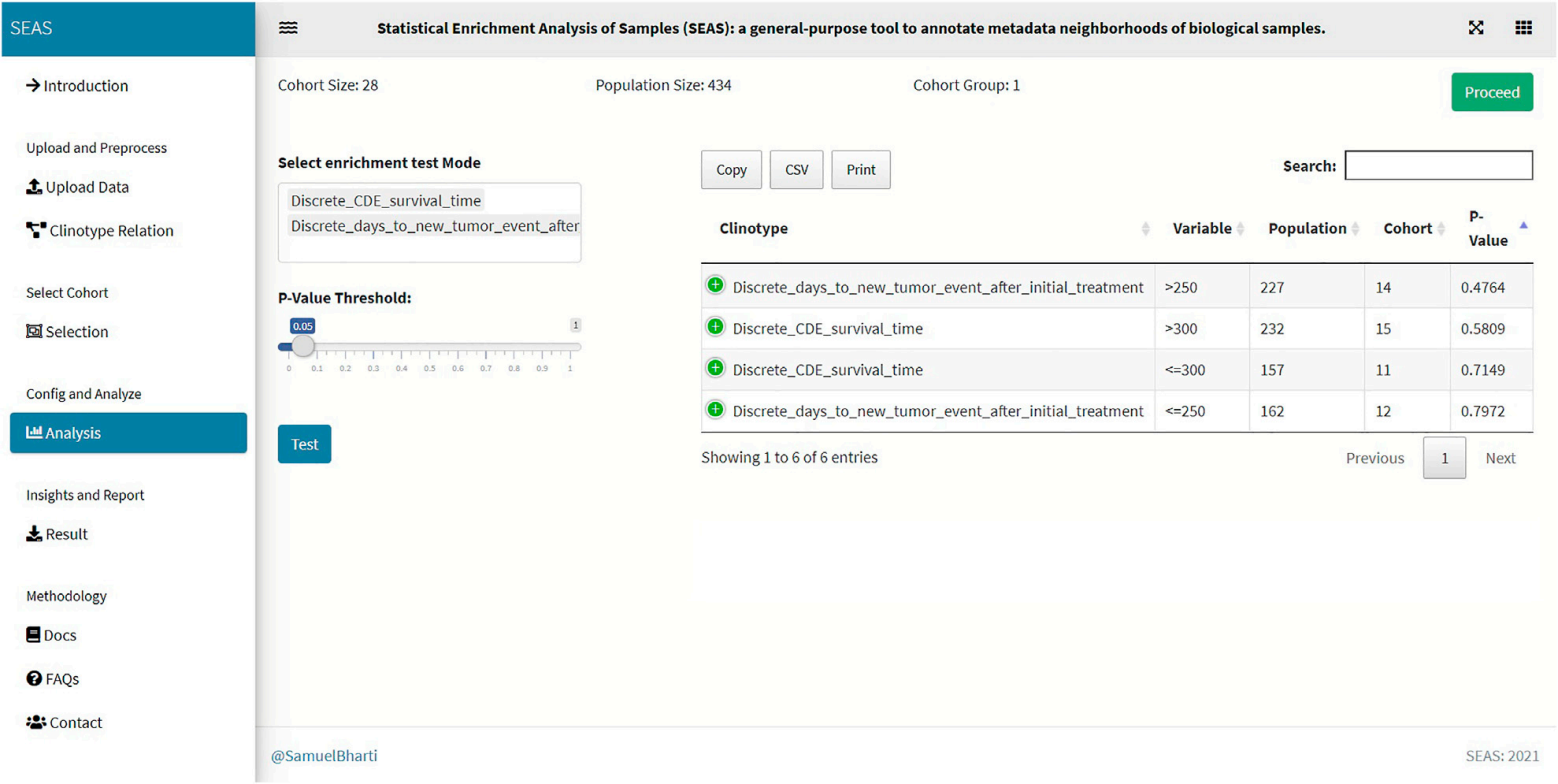
SEAS identifies enriched clinical features for the subcohort in Figure 4.

**FIGURE 6:**
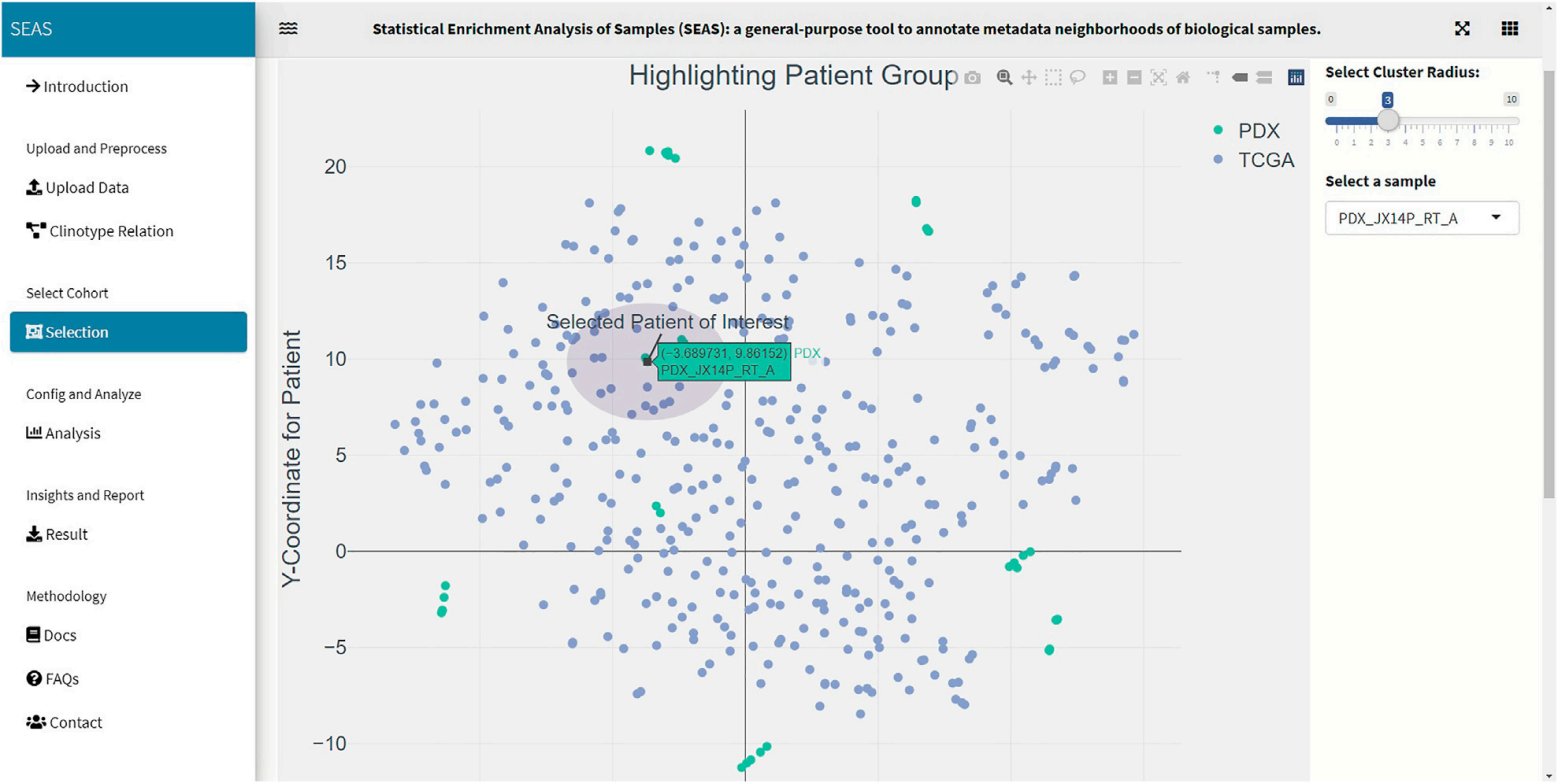
SEAS identifies a subcohort by a circle region around PDX JX14P_RT_A datapoint.

**FIGURE 7:**
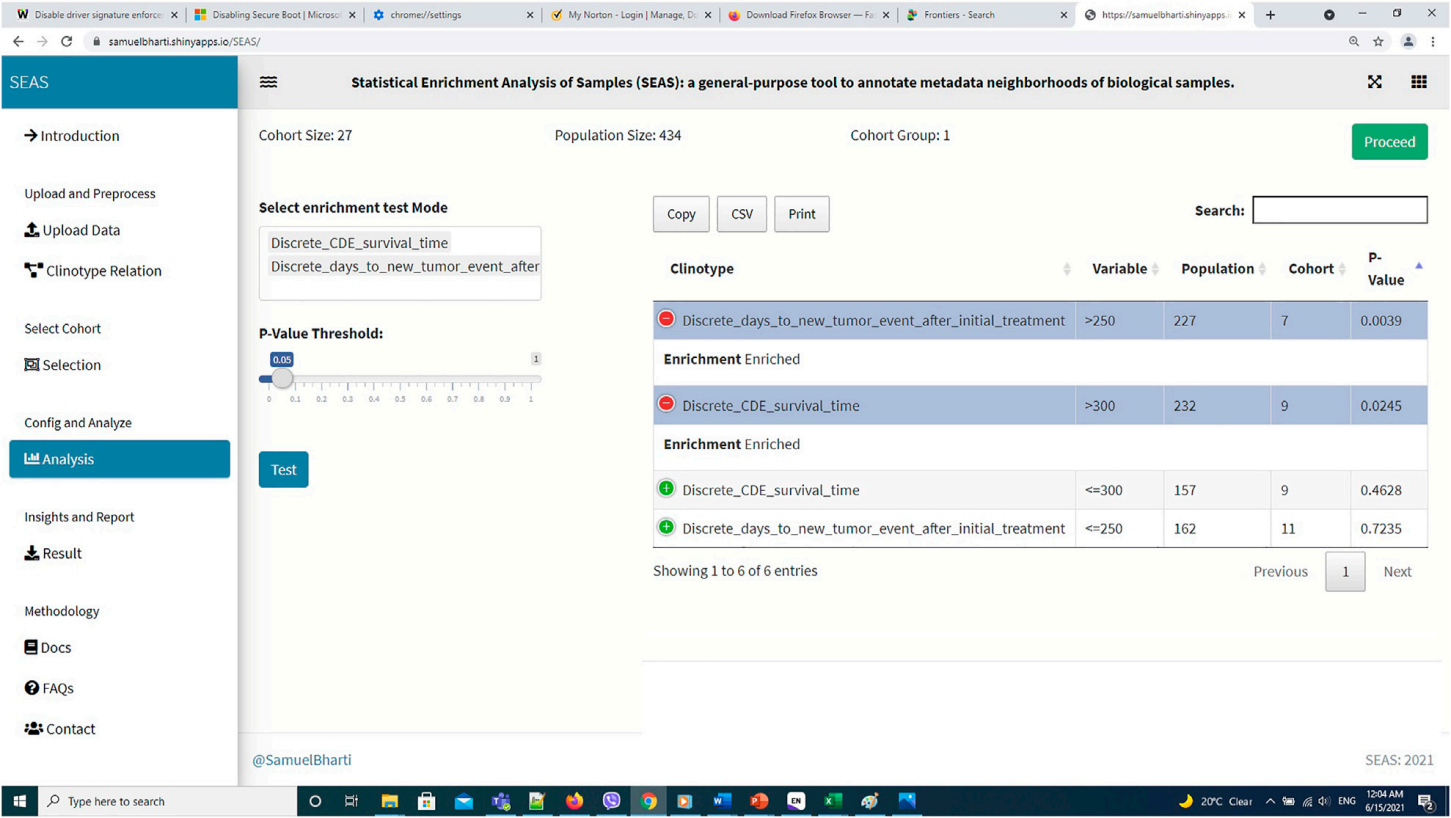
SEAS identifies enriched clinical features for the subcohort in Figure 6.

After digitizing the clinical attributes, SEAS applies the embedding method (Figures 2–7) to represent the patients in a 2D space. By default, SEAS uses the umap (McInnes et al., 2018) algorithm. Alternatively, the user may also select tSNE (Hinton and Roweis, 2002) for embedding. SEAS computes patients’ similarities using the 2D embedded coordinate.

### Automatically Select a Subcohort

In SEAS, the user can manually define a subcohort by typing the list of patient IDs (Figure 2). Besides, the user may use SEAS to automatically select a subcohort in two ways. In the fully automatic approach, SEAS applies clustering algorithms to divide the patient data into multiple groups. Then, the user selects a group as a subcohort. This approach is preferred because the clustering results can provide the threshold to discretize the numerical attributes into categorical attributes, resulting in the next step. By default, SEAS uses the density-based clustering algorithm (Ester et al., 1996, Figure 3). In the semi-automatic approach (Figures 4, 6), the user selects a patient ID, a radius of “similarity area” in the 2D embedding space. All patients in the circle area are centered by the selected patient ID, and the radius becomes the selected subcohort.

### Analyze Clinical Feature Enrichment

Besides implementing Wilcoxon-ranksum (Mann and Whitney, 1947) and test between the selected cohort and the whole population for numerical attributes, SEAS defines the CFEA that can be applied for both numerical and categorical attributes. Here, we denoted a patient population **S** and a set of all clinical attributes **C**. Given any cohort **s** in **S**, the main question is which attributes are representative or enriched in **s**. For a categorical attribute, SEAS applies the hypergeometric test, which compares the proportions of patients having the attribute between **s** and **S**. This approach is well-known in gene set enrichment analysis (Falcon and Gentleman, 2008). Here, the null hypothesis is the proportion of patients having attribute C in **s** and **S** is the same. This is analog to the null hypothesis in the Wilcoxon-ranksum (Mann and Whitney, 1947) test, where the median of attribute **C** in **s** and **S** is the same. To apply in numerical data, the numerical attributes are discretized. For example, in our GBM case study, “CDE_survival_time” (survival day), which is a numerical attribute, is discretized into “Discrete_CDE_survival_time <300 days” and “Discrete_CDE_survival_time ≥300 days.” As mentioned in the previous section, clustering the patient and using the cluster to determine the numerical thresholds is a good approach. SEAS reports all enriched clinical attributes and their *p*-values and the Bonferroni adjusted *p*-value (for false discovery rate control) (Sedgwick, 2014), as in Figure 5.

### Implementing the Software

The SEAS web version is built primarily by bs4Dash (https://cran.r-project.org/web/packages/bs4Dash/index.html) and R-shiny (https://shiny.rstudio.com/) packages. Both packages run based on R and can be hosted inside well-known web programming languages: HTML, CSS, and javascript. In addition, the data processing and statistical methods are also implemented in R.

### Demo Using TCGA-GBM Dataset

We acquired and preprocessed TCGA-GBM dataset, which consists of 389 patients, according to the pipeline in Jia et al. (2018). The dataset had both the genetic and the clinical sections. Among 108 clinical attributes, 22 categorical and seven numerical ones were used to compute patient similarity and embedding (Supplementary Data S1). Also, we used 45 GBM tumor-samples hosted in patient-derived xenograft (PDX) models (Willey et al., 2020). In these samples, the patients were treated by radiation therapy (RT), but did not have clinical information. Besides the automatic embedding using the clinical data, we manually applied tSNE (Hinton and Roweis, 2002) on the combined TCGA-GBM and PDX genetic data as another 2D representation. We checked the quality of the embedding by the close positions of the PDX JX14P_A/JX14P_B sample pair and the PDX JX14P_RT_A/JX14P_RT_B sample pair. These pairs are replicates of the same patient tumor JX14P (before radiation therapy) and JX14P_RT (after radiation therapy—RT), as shown in Supplementary Figure S1.

In this case study, to estimate the clinical outcome of an unknown PDX sample, we select a TCGA-GBM subcohort surrounding the PDX sample (Figures 4, 6) and performed SEAS in the selected TCGA subcohort. In Figures 4, 5, SEAS shows no enriched clinical feature for sample PDX JX14P_A. Here, the average survival time among the surrounding TCGA patients was 339 days. In Figures 6, 7, feature “Discrete_CDE_survival_time >300”, which means that the patients who survive for more than 300 days, are enriched among the TCGA samples surrounding the PDX JX14P_RT_A sample. Here, the average survival time for these patients was 434 days. This result suggests radiation therapy may improve the clinical condition of the JX14P patient. Thus, SEAS analysis suggests two opposite clinical outcomes for GBM patients even when being treated by the same therapy. The finding could be helpful in further clinical decisions regarding the selected patients.

### Other Notes About Similarity Measures and Embedding Options

#### Similarity Measures

In SEAS, we used the embedded coordinates to compute the Euclidean distance between two patient datapoints

$$d\left(i,j\right)=\sqrt{{\left(x_{i}-x_{j}\right)}^{2}+{\left(y_{i}-y_{j}\right)}^{2}}$$ (1)

Here, *i* and *j* denotes two patients, d(i,j) denotes the distance between *i* and *j*, ( $x_{i},\;y_{i}$ ) denotes the embedded coordinate for patient *i*, and ( $x_{j},\;y_{j}$ ) denotes the embedded coordinate for patient *j*. We did not use any other similarity measure because we assume that the good embedding results already reflect the patient-wise similarity. In case the user’s defined similarity could not be reflected by SEAS, the user can manually enter the list of similar patients to perform the enrichment analysis.

#### Embedding Options

By default, if the user does not supply the embedding input, SEAS may use umap (McInnes et al., 2018) or tSNE (Hinton and Roweis, 2002) to embed the patient from the clinical features. The embedding algorithms, as in (Konopka, 2020), require a pairwise distance or similarity matrix. At this release, SEAS supports the Euclidean distance (default), cosine similarity, and Jaccard index. Besides, the user is encouraged to supply an embedding file for more in-depth analysis. For example, in our GBM case study, the patient pairwise similarity and embedding are computed by the gene expression data instead of the clinical feature. The PDX have gene expression data but do not have clinical attributes; therefore, they could not be embedded correctly with SEAS default option. When the clinical data is insufficient to compute good embedding results, we highly recommend the user to use other tools to compute the embedding prior to using SEAS.

## Discussion and Conclusion

To summarize, we developed the user-friendly and online version of SEAS. The tool can provide new and significant insights into clinical data research and may support the clinical decision. In the future, we expect to develop the add-on version of SEAS, which can be integrated into I2B2 clinical data management system.

One limitation in this SEAS first release is that we have not implemented techniques handling missing values in the patients’ clinical data. To lower the impact of this limitation, we chose the enrichment methods, such as the hypergeometric test, that do not require a very large data size. In our GBM case study, the population consists of 389 patients, which is a moderate size. However, it is sufficient to perform the statistical test even if the missing data rate for one clinical attribute is 10%. On the other hand, we encourage the user to use the non-clinical data to embed the patients; therefore, the missing clinical data may not impact the quality of SEAS results. In fact, our GBM case study shows an approach to infer unknown clinical attributes in PDX data by SEAS analysis of TCGA-GBM data.

## Data Availability

Publicly available datasets were analyzed in this study. This data can be found here: https://github.com/aimed-uab/SEAS.

## Supplementary Material

Supplementary Figure S1 Locations of datapoint pairs PDX JX14P_A / JX14P_B sample pair (top) and the PDX JX14P_RT_A / JX14P_RT_B (bottom).
