# Corrigendum: Statistical Enrichment Analysis of Samples: A General-Purpose Tool to Annotate Metadata Neighborhoods of Biological Samples

**DOI:** 10.3389/fdata.2021.804141

**Published:** 2021-11-16

**Authors:** Thanh M. Nguyen, Samuel Bharti, Zongliang Yue, Christopher D. Willey, Jake Y. Chen

**Affiliations:** ^1^ Informatics Institute, School of Medicine, The University of Alabama at Birmingham, Birmingham, AL, United States; ^2^ Centre for Computational Biology and Bioinformatics, Amity Institute of Biotechnology, Amity University, Noida, India; ^3^ Department of Radiation Oncology, School of Medicine, The University of Alabama at Birmingham, Birmingham, AL, United States

**Keywords:** sample enrichment analysis, clinotype, SEAS, glioblastoma multiforme, patient-derived xenograft

In the original article, we neglected to include the **Acknowledgments**. The **Acknowledgments** appears below.

“All authors thank the following general technical support that made case studies included for this work possible: Christian Stackhouse for helping CW generate the GBM-PDX model, Jelai Wang for managing the data management and data analysis computing framework, and Christian Stackhouse and Lara Lanov for executing the RNA-seq analysis pipelines.”

The authors apologize for this error and state that this does not change the scientific conclusions of the article in any way. The original article has been updated.

