# Supplementary material for: Statistical Enrichment Analysis of Samples: A General-Purpose Tool to Annotate Metadata Neighborhoods of Biological Samples
Source: Front Big Data. 2021 Sep 16;4:725276. doi: 10.3389/fdata.2021.725276 (PMC8481385; doi:10.3389/fdata.2021.725276)
Supplement: Supplementary file 1 [file Presentation1.pdf]

SEAS overview

In this document, we present

- SEAS purpose

- SEAS session workflow

- Input and output format

- Navigating through SEAS

- Current technical limitation

- How to contribute to SEAS

I. SEAS purpose

Statistical Enrichment Analysis of Samples (SEAS) is a tool to find which clinical (metadata) attributes are enriched within a sample subset. For example, SEAS answer the following questions:

- I have population data with brain cancer survival time; I select an interested patient subcohort, such as who received X treatment; does this subcohort have long survival time?

SEAS can be used to infer or annotate the unknown clinical (metadata) attribute of a sample. For example:

- I same a brain cancer patient whom I do not know the survival time; can I use SEAS to infer the survival time of the patient?

To do so, I can define a subcohort, which includes the most similar patients to the unknown survival-time patient. The question is converted to the one above, which can be answered by SEAS. Also, in SEAS, I can use embedding to view similar patients.

II. SEAS session workflow

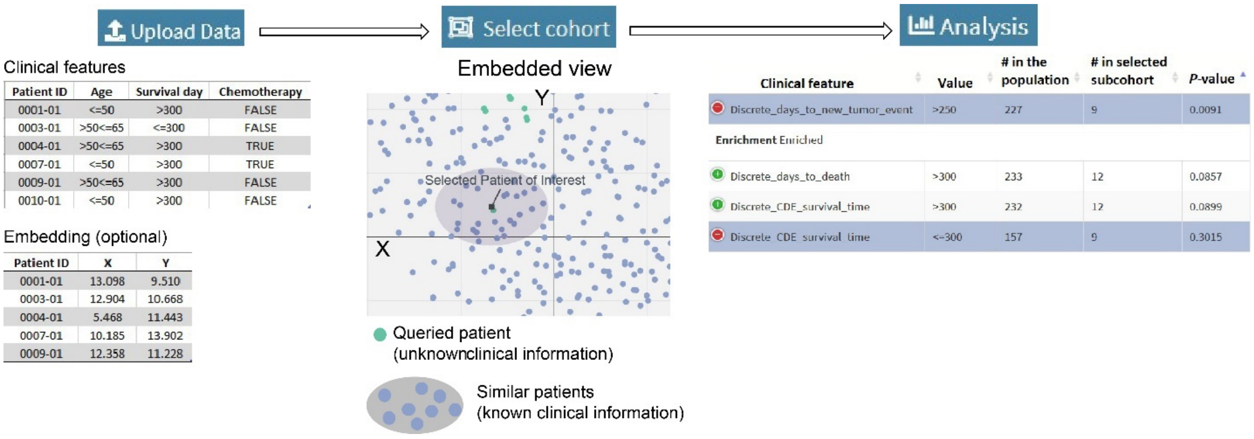

Figure 1. Overview of a SEAS session

As showed in Figure 1, a SEAS session (https://aimed-lab.shinyapps.io/SEAS/) includes three steps:

- Uploading data: the user upload the clinical metadata for each sample (required) and/or the embedding for these samples (optional)

The purpose of embedding is to visualize the similarity among the samples. Each sample is represented by a 2D point. The closer the two points are, the more similar the two samples are.

- Selecting cohort: the user can manually select an interested subcohort or use the embedding to select a subcohort where the samples are similar to each other.

- Analyze: the result shows which clinical attributes are enriched (dominant) in the selected subcohort. For each attribute, its the p-value tells, statistically, how likely the attribute are enriched. By default, if the p-value is less than 0.05, the attribute is considered significantly enriched.

The functional workflow is as follow

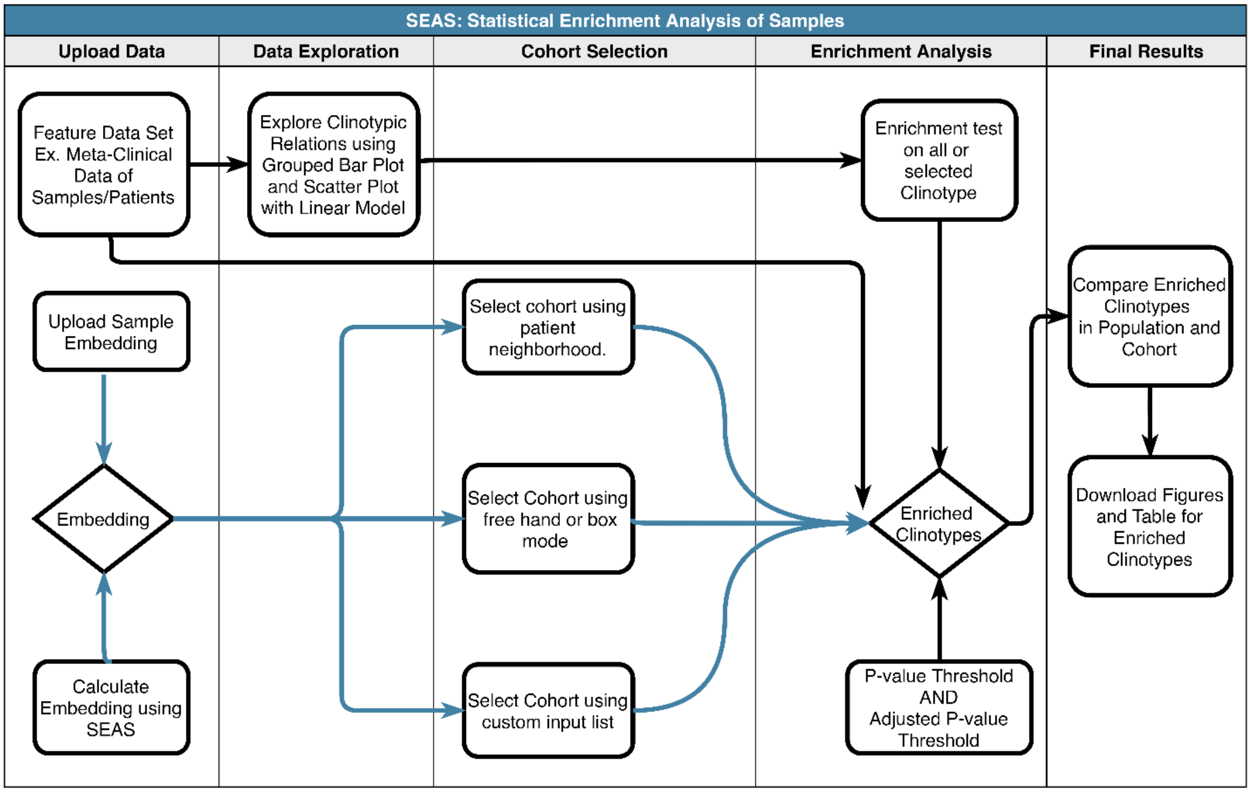

III. Input and format

The input files for SEAS are in table text format. The table column can be separated by 'tab' or 'comma'. 'Tab' is more preferred. Excel is recommended to prepare the input text file.

- The clinical table first column should be the sample identifier (i.e. patient ID). An example of the clinical table format is as follow:

Patient ID	Age	Survival day	Chemotherapy
0001-01	<=50	>300	FALSE
0003-01	>50<=65	<=300	FALSE
0004-01	>50<=65	>300	TRUE
0007-01	<=50	>300	TRUE
0009-01	>50<=65	>300	FALSE
0010-01	<=50	>300	FALSE

- The embedding table only has three columns: the sample identifier, the embedding x-coordinate of these samples, and the embedding y-coordinate of these samples. An example of the clinical table format is as follow:

Patient ID	X	Y
0001-01	13.098	9.510
0003-01	12.904	10.668
0004-01	5.468	11.443
0007-01	10.185	13.902
0009-01	12.358	11.228

The sample identifier order between the two tables should match.

IV. Navigating through SEAS

1. Embedding

Embedding is a key element for SEAS to have good results. The user may choose either tSNE or umap algorithm to embed the sample if the user does not prepare the embedding input file. Still, we encourage the user to prepare and examine the embedding before analyzing using SEAS carefully.

2. Exploring data (optional)

SEAS allows users to visualize the clinical feature relations through grouped bar plots and scatter plots. upon uploading the dataset

SEAS automatically identifies the data type of each clinotype in the dataset and places them in respective suitable plots.

Linear Model Prediction is also added inside the scatter plot to visualize the correlation between two clinotypes.

3. Subcohort selection

SEAS support the following ways to select the subcohort:

- Box selection: the user draw a bounding box that covers some samples in the embedding visualization. SEAS would recognize the samples inside the box as the subcohort.

- Neighbor-point selection: in the embedding visualization, the user chooses a sample as the center and a radius. This defines a circle. SEAS would recognize all sample points inside the circle as the subcohort.

- Entering sample selection: the user can enter the list of sample identifiers into a box to define a subcohort.

4. Understanding the result

SEAS presents the enrichment result in a table, typically as follow

Clinical feature	Value	# in the population	# in selected subcohort	*P*-value
Discrete_days_to_new_tumor_event	>250	227	9	0.0091
**Enrichment** Enriched				
Discrete_days_to_death	>300	233	12	0.0857
Discrete_CDE_survival_time	>300	232	12	0.0899
Discrete_CDE_survival_time	<=300	157	9	0.3015

- The first column is the feature name. The second feature is the value. For example, the figure about shows ‘Discrete_days_to_death > 300‘ (outcome: the patient survive for more than 300 days)

- # in the population: the number of samples that have clinical outcomes defined by the previous two columns in the whole population.

- # in the selected cohort: the number of samples that have the clinical outcome defined by the previous two columns in the selected subcohort

- p-value: the result of statistical test for clinical enrichment. The smaller p-value is, the more likely the clinical outcome is prevalent in the selected subcohort.

V. Current technical limitations

- The current SEAS version is deployed in an online machine where the memory allocation is only 2GB. Therefore, we recommend that the input file size should be less than 100 MB. This input size usually has less than 10000 samples.

- The user may see the error, which says, ‘An error has occurred. Check your logs or contact the app author for clarification’. We have investigated these issues and found that the issues are not related to our implementation. Two reasons for these issues are:

+ Long time without interaction. Usually, the SEAS online tool would return an error if the user does not interact with SEAS within 3-5 minutes

+ System slow computation and response. That is, the user interacts and expects some visualization (i.e. embedding plot) while the system has not yet computed and processed.

To completely solve these issues, we may upgrade the SEAS server. This requires a monthly payment to shinyapps.io. Due to the financial processing time requirement, we have not yet completed the paperwork for the upgrade. Meanwhile, the user may try deploying SEAS code at shinyapps.io inside an in-house computer.

VI. How to contribute to SEAS

We welcome the user's feedback and contributed dataset for future SEAS development. Please email SEAS developer the issues and sample dataset at:

jakechen@uab.edu (Jake Chen, supervisor)

thamnguy@uab.edu (Thanh Nguyen, the architect)

sbharti@uab.edu (Samuel Bharti, the programmer).
