# Supplementary figures and images for: Statistical Enrichment Analysis of Samples: A General-Purpose Tool to Annotate Metadata Neighborhoods of Biological Samples
Source: Front Big Data. 2021 Sep 16;4:725276. doi: 10.3389/fdata.2021.725276 (PMC8481385; doi:10.3389/fdata.2021.725276)

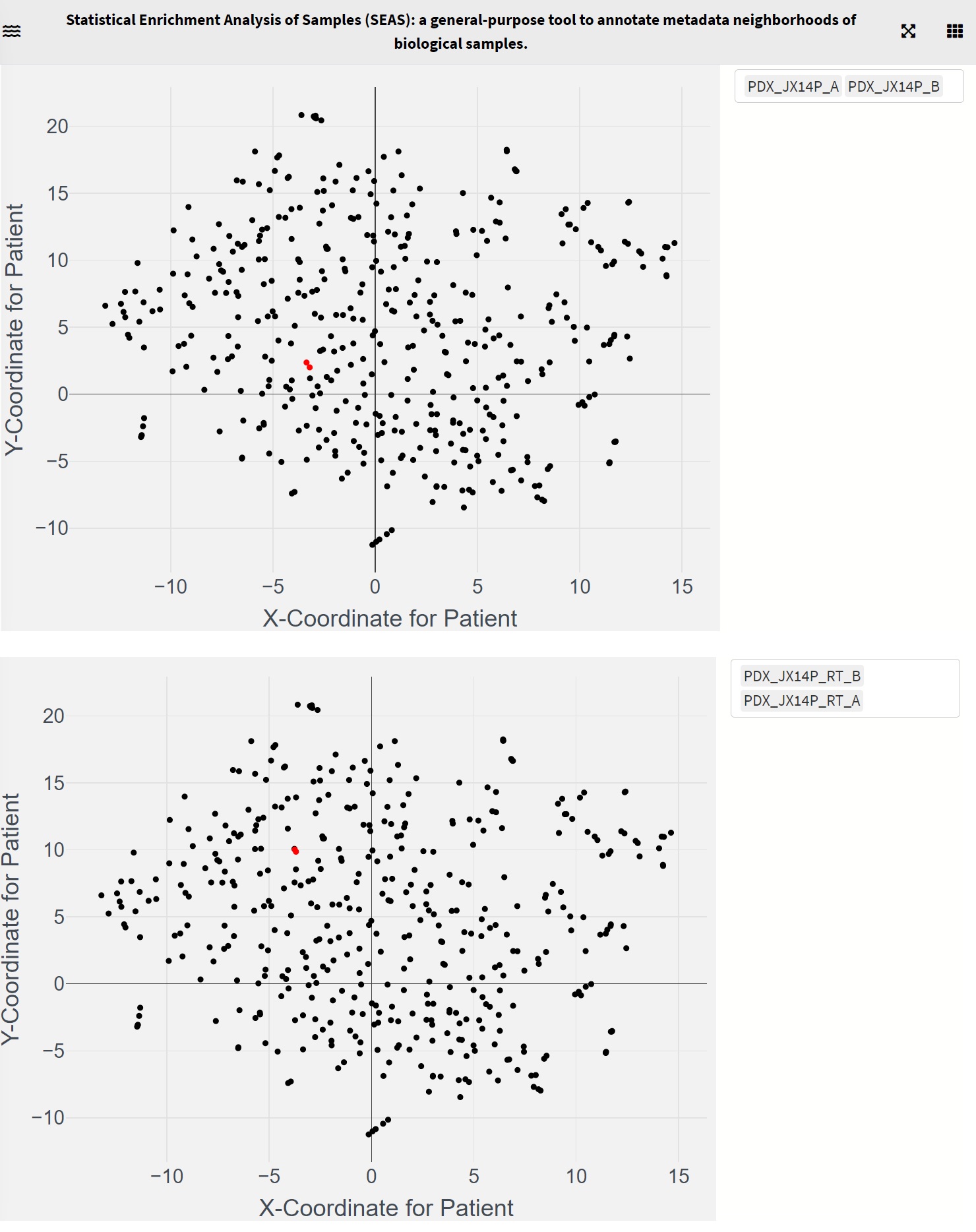

Supplement: Supplementary file 2 [file Image1.JPEG]
