# Supplementary material for: Statistical Enrichment Analysis of Samples: A General-Purpose Tool to Annotate Metadata Neighborhoods of Biological Samples
Source: Front Big Data. 2021 Sep 16;4:725276. doi: 10.3389/fdata.2021.725276 (PMC8481385; doi:10.3389/fdata.2021.725276)
Supplement: Supplementary file 5 [file Presentation2.pdf]

SEAS Tutorial

1. Data Importation.

   SEAS require two data inputs from the user:

   a. Meta-Clinical data of Patients.
   b. Embedding of Patients/Samples

   First input is the meta-clinical data of patients with n columns. Patient Identifier should be placed in first column. Values of first column should be identical in both the input files. **Embedding of Patients** can be uploaded in a csv format with 3 column data set. First column is the Patient Identifier, consecutive columns are X and Y coordinates of embedding. User can also choose select the option to calculate embedding directly through SEAS by selecting either tSNE or UMAP algorithm for the processed meta-clinical data set.

   Accepted File Format: ".csv"

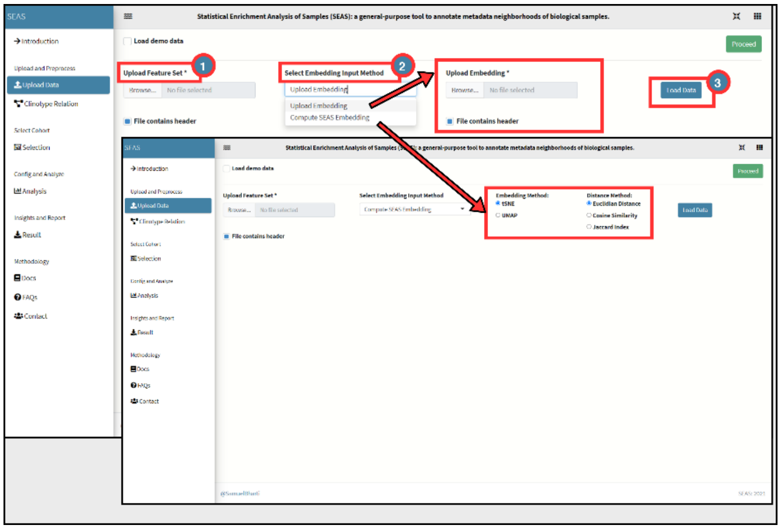

   For demo of SEAS:

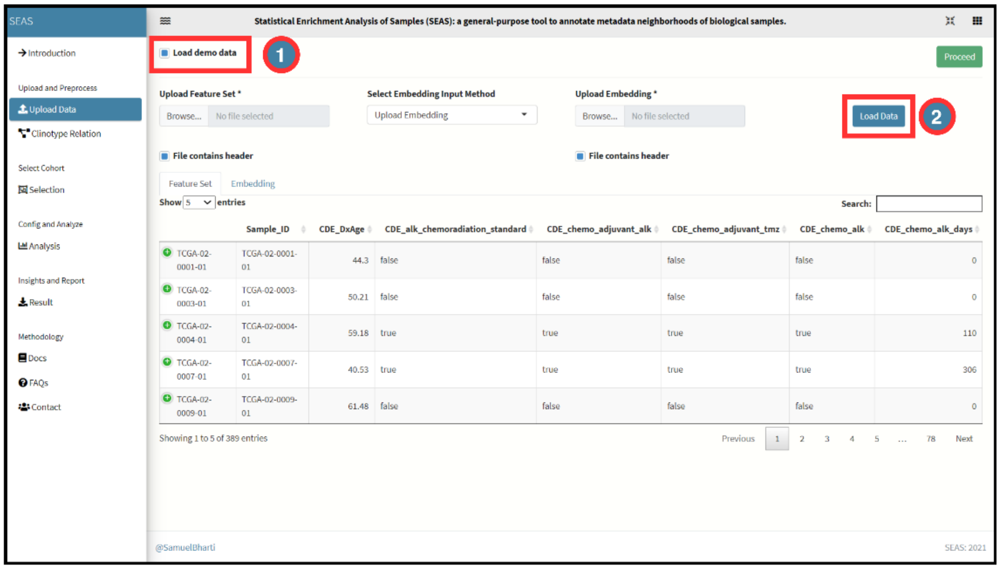

2. Data Exploration
   Upon uploading of dataset, SEAS allows user to visualize the clinotypic (patient features) relations through grouped bar plots and scatter plots.
   SEAS automatically identifies the data type of each clinotype in dataset and places them in respective suitable plots.
   Linear Model Prediction is also added inside the scatter plot to visualize correlation between two clinotypes.

3. Cohort Selection
   SEAS consist of three different methods for cohort selection from the embedding plot. Different methods provides user more flexibility in picking a cohort from a neighborhood or distantly located places depending on the hypothesis of experiment.

   a. Lasso + Box Selection Method.
      The method allows user to either free hand or box select multiple clusters of patients/samples from the embedding plot.

   b. Neighborhood Selection Method.
      The method allows user to select a patient of interest from the dropdown and then select a neighborhood (closely located) using a dynamic radius input. This method is useful ___".

   c. Custom Selection Method.
      The method allows user to input or provide custom list of Patient Identifiers to select in the embedding plot. This method is useful when the user selects a cohort with prior knowledge either from clinical or genomic profiles of patients.

4. Enrichment Analysis
   After successfully selecting a cohort, the user can now statistically analyze enrichment in cohort clinotypes comparing with population clinotypes.

   SEAS allows user to select a clinotype(s) of interest to test or simply select first option "All" to test whole dataset.

   User can also set the P-value threshold for the test or leave on default to 0.05.

   A table with detailed information of results is shown to the right. Calculated values are p-value and adjusted p-value using FDR cutoff of 0.05.

5. Result
   Clinotypes which passed through the p-value threshold are shown here to further visualize the cohort and background relation using pie chart and boxplot.
   Each row in this table is clickable and simultaneously updates the visualization plots of selected/clicked clinotype.
